# 1-(4-Nitro­phen­yl)-1*H*-imidazol-3-ium chloride

**DOI:** 10.1107/S1600536812050878

**Published:** 2012-12-19

**Authors:** Halliru Ibrahim, Muhammad D. Bala

**Affiliations:** a School of Chemistry & Physics, University of KwaZulu-Natal, Westville Campus, Private Bag X54001, Durban 4000, South Africa

## Abstract

In the title salt, C_9_H_8_N_3_O_2_^+^·Cl^−^, the least-squares planes of the imidazolium and benzene rings are almost coplanar, making a dihedral angle of 4.59 (1)°. In the crystal, the chloride anion links the organic mol­ecules through N—H⋯Cl hydrogen bonds, forming chains that run diagonally across the *bc* face, which compliment strong C—H⋯O hydrogen bonds between neighbouring mol­ecules. These chains are connected to adjacent chains through two weak C—H⋯Cl inter­actions, resulting in hydrogen-bonded sheets extending along the *b* and *c* axes. The absolute structure of the title compound was determined using a Flack *x* parameter of 0.00 (6) and a Hooft *y* parameter of 0.03 (2).

## Related literature

For the synthesis of the title compound, see: Gnanamgari *et al.* (2009 ▶); Coberan & Peris (2008 ▶); Singh *et al.*, (2011 ▶). For the structure of imidazole with a bond to phenyl *via* carbon, see: Gayathri *et al.* (2010 ▶). For structure of imidazole with a bond to phenyl *via* nitro­gen, see: Zheng *et al.* (2011 ▶). For the structure of nitro­phenyl imidazole as a ligand in a complex, see: Singh *et al.* (2010 ▶, 2011 ▶). For related structures, see: Ishihara *et al.* (1992 ▶); Scheele *et al.*, (2007 ▶). For our related work in this area, see: Ibrahim *et al.* (2012 ▶).
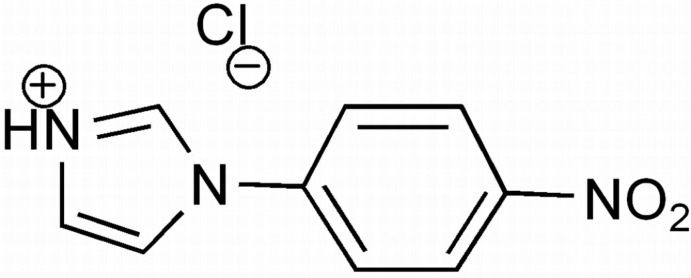


## Experimental

### 

#### Crystal data

C_9_H_8_N_3_O_2_^+^·Cl^−^
*M* *_r_* = 225.64
Orthorhombic, 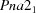

*a* = 14.6042 (8) Å
*b* = 12.1781 (7) Å
*c* = 5.6070 (3) Å
*V* = 997.21 (10) Å^3^
*Z* = 4
Mo *K*α radiation
μ = 0.37 mm^−1^
*T* = 173 K
0.54 × 0.16 × 0.15 mm

#### Data collection

Bruker SMART APEXII CCD diffractometer
Absorption correction: multi-scan (*SADABS*; Bruker, 2008 ▶) *T*_min_ = 0.524, *T*_max_ = 0.746
20153 measured reflections
2217 independent reflections
2120 reflections with *I* > 2σ(*I*)
*R*_int_ = 0.060

#### Refinement

*R*[*F* ^2^ > 2σ(*F* ^2^)] = 0.029
*wR*(*F* ^2^) = 0.076
*S* = 1.09
2217 reflections
140 parameters
8 restraints
H atoms treated by a mixture of independent and constrained refinement
Δρ_max_ = 0.30 e Å^−3^
Δρ_min_ = −0.22 e Å^−3^
Absolute structure: Flack (1983 ▶), Hooft *et al.* (2010 ▶), Spek (2009 ▶); Hooft parameter = 0.03 (2), 856 Bijvoet pairs
Flack parameter: 0.00 (6)

### 

Data collection: *APEX2* (Bruker, 2008 ▶); cell refinement: *SAINT-Plus* (Bruker, 2008 ▶); data reduction: *SAINT-Plus* and *XPREP* (Bruker, 2008 ▶); program(s) used to solve structure: *SHELXS97* (Sheldrick, 2008 ▶); program(s) used to refine structure: *SHELXL97* (Sheldrick, 2008 ▶); molecular graphics: *ORTEP-3* (Farrugia, 2012 ▶); software used to prepare material for publication: *WinGX* (Farrugia, 2012 ▶).

## Supplementary Material

Crystal structure: contains datablock(s) global, I. DOI: 10.1107/S1600536812050878/nr2034sup1.cif

Structure factors: contains datablock(s) I. DOI: 10.1107/S1600536812050878/nr2034Isup2.hkl

Supplementary material file. DOI: 10.1107/S1600536812050878/nr2034Isup3.cml

Additional supplementary materials:  crystallographic information; 3D view; checkCIF report

## Figures and Tables

**Table 1:** Hydrogen-bond geometry (Å, °)

*D*—H⋯*A*	*D*—H	H⋯*A*	*D*⋯*A*	*D*—H⋯*A*
N1—H1⋯Cl1^i^	0.92 (2)	2.08 (2)	2.9976 (17)	178 (2)
C9—H9⋯Cl1	0.93	2.80	3.5898 (19)	144
C2—H2⋯Cl1^ii^	0.93	2.52	3.4286 (17)	166
C4—H4⋯O2^i^	0.93	2.29	3.181 (2)	161

Symmetry codes: (i) 
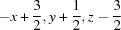
; (ii) 
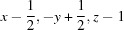
.

**Fig. 1.**
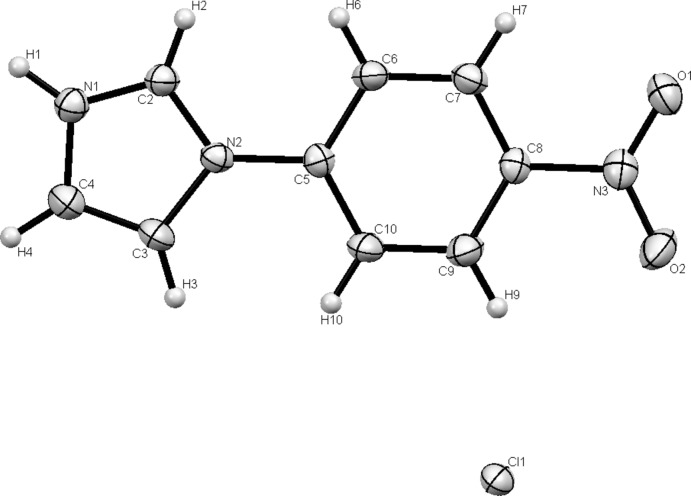
ORTEP diagram of compound (I). Thermal ellipsoids are represented at the 50% probability level.

**Fig. 2.**
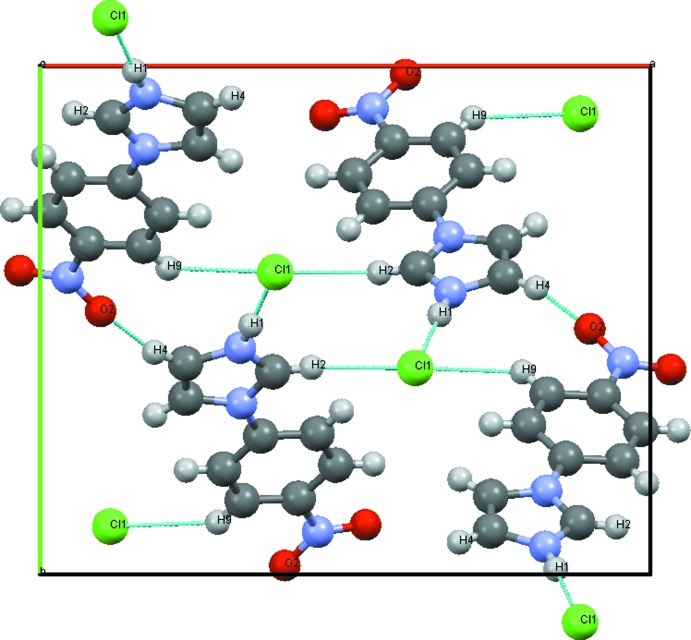
Packing diagram showing hydrogen bonding interactions in a crystal of (I) viewed along crystallographic c axis.

## supplementary crystallographic information

### Comment

Since the isolation of the first stable free carbene, imidazolium based *N*-heterocyclic carbene ligands (NHC) ligands have recieved wide interest from researchers because substituted imidazolium salts are major precursors to the NHCs commonly employed in organometallic chemistry and catalysis for the stabilization of metal centers. Recently Gayathri *et al.*, (2010) have reported structural analogues of the title compound with imidazole bond to phenyl *via* carbon, while Zheng *et al.*, (2011) have reported the structure with imidazole bond to phenyl *via* nitrogen. For the structure of nitrophenyl imidazole as a ligand in a metal complex, see: (Singh *et al.*, 2010 and 2011). Structures of related compounds were reported by Ishihara *et al.*, (1992), Scheele *et al.*, (2007) and Ibrahim *et al.*, (2012). Hence, the title compound was obtained in an attempt to synthesize an imidazolium salt by the coupling of 2-chloromethylpyridine hydrochloride with *p*-nitrophenyl imidazole using the method reported by Gnanamgari *et al.*, (2009). Coberan & Peris (2008) and Singh *et al.*, (2011) have also reported synthesis of similar compounds. The grey solid obtained was recrystallized from methanol:ethyl acetate (1:1) solvent system. The planes of the imidazolium and phenyl rings in (I) are almost coplanar. Analysis of the absolute structure using likelihood methods (Hooft *et al.*, 2010) was performed using *PLATON* (Spek, 2009). The Hooft *y*-parameter was determined to be 0.03 (2) which corroborated the Flack parameter *x* = 0.00 (6). These results in conjunction with a correlation coefficient of 0.997 for the Bijvoet normal probability plot indicate that the absolute structure is correctly assigned. In the title compound, C_9_H_8_N_3_O_2_.Cl, the L.S. planes of the imidazolium (N1—C4) and phenyl (C5—C10) rings are almost coplanar with a dihedral angle of 4.59 (1)°. In the crystal, the chloride atom links the organic molecules through N—H···Cl hydrogen bonds forming chains that run diagonally across the *bc* face which compliment strong intermolecular C—H···O hydrogen bonds between neighbouring molecules. These chains are connected to adjacent chains through two weak C—H···Cl interactions resulting in hydrogen bonded sheets extending along the b and c axes.

### Experimental

To a 150 ml round bottom flask containing DMSO (30 ml, MERCK) was added imidazole (0.01 mol, 0.68 g, Fluka AG) and KOH (0.015 mol, 0.84 g, MERCK) then stirred at room temperature for 2 h. This was followed by the dropwise addition of a solution of 1-chloro-4-nitrobenzene (Fluka, 0.01 mol, 1.57 g) in DMSO (5 ml), and refluxed at 100 °C for 24 h. The resulting solution was first chilled and then dilute with distilled water until neutral. The organic component was extracted using CH_2_Cl_2_/CHCl_3_ (1:1, 3 *x* 20 ml) and then dried with anhydrous MgSO4 and concetrated under vacuum yielding 2.081 g of pure (I). ^1^H NMR (400 MHz, CDCl_3_): 8.36(d; 2H) 7.96(s; 1H), 7.57(d; 2H) and 7.25(1*H*) p.p.m.. ^13^C NMR (400 MHz, CDCl_3_): 146.6, 142.3, 135.7, 132.04, 126.1, 121.4 and 117.9 p.p.m.. IR (ATR): 3112(=C—H), 2924(*sp*^3^ C—H), 1596(C=N), 1503 and 1370(aromatic NO_2_), 1049 (C—N medium) and 845 (*p*-subsituted benzene) cm^-1^.

### Refinement

Carbon-bound H-atoms were placed in calculated positions [C—H = 0.93 Å for aromatic H atoms; *U*_iso_(H) = 1.2*U*_eq_(C)] and were included in the refinement in the riding model. The nitrogen-bound H atom was located on a difference Fourier map and refined freely with isotropic parameters.

### Crystal data

**Table:**

C_9_H_8_N_3_O_2_^+^·Cl^−^	*F*(000) = 464
*M**_r_* = 225.64	*D*_x_ = 1.503 Mg m^−^^3^
Orthorhombic, *P**n**a*2_1_	Mo *K*α radiation, λ = 0.71073 Å
Hall symbol: P 2c -2n	Cell parameters from 9896 reflections
*a* = 14.6042 (8) Å	θ = 2.2–28.3°
*b* = 12.1781 (7) Å	µ = 0.37 mm^−^^1^
*c* = 5.6070 (3) Å	*T* = 173 K
*V* = 997.21 (10) Å^3^	Block, colourless
*Z* = 4	0.54 × 0.16 × 0.15 mm

### Data collection

**Table:**

Bruker SMART APEXII CCD diffractometer	2217 independent reflections
Radiation source: fine-focus sealed tube	2120 reflections with *I* > 2σ(*I*)
Graphite monochromator	*R*_int_ = 0.060
φ and ω scans	θ_max_ = 28.3°, θ_min_ = 2.2°
Absorption correction: multi-scan (*SADABS*; Bruker, 2008)	*h* = −17→19
*T*_min_ = 0.524, *T*_max_ = 0.746	*k* = −16→16
20153 measured reflections	*l* = −7→6

### Refinement

**Table:**

Refinement on *F*^2^	Secondary atom site location: difference Fourier map
Least-squares matrix: full	Hydrogen site location: inferred from neighbouring sites
*R*[*F*^2^ > 2σ(*F*^2^)] = 0.029	H atoms treated by a mixture of independent and constrained refinement
*wR*(*F*^2^) = 0.076	*w* = 1/[σ^2^(*F*_o_^2^) + (0.0354*P*)^2^ + 0.3302*P*] where *P* = (*F*_o_^2^ + 2*F*_c_^2^)/3
*S* = 1.09	(Δ/σ)_max_ < 0.001
2217 reflections	Δρ_max_ = 0.30 e Å^−^^3^
140 parameters	Δρ_min_ = −0.22 e Å^−^^3^
8 restraints	Absolute structure: Flack (1983), Hooft *et al.* (2010) and Spek (2009); Hooft parameter = 0.03(2), 856 Bijvoet pairs
Primary atom site location: structure-invariant direct methods	Flack parameter: 0.00 (6)

### Special details

**Table:**

Experimental. Carbon-bound H-atoms were placed in calculated positions [C—H = 0.93 Å for aromatic H atoms; *U*_iso_(H) = 1.2*U*_eq_(C)] and were included in the refinement in the riding model. The nitrogen-bound H atom was located on a difference Fourier map and refined freely with isotropic parameters.
Geometry. All e.s.d.'s (except the e.s.d. in the dihedral angle between two l.s. planes) are estimated using the full covariance matrix. The cell e.s.d.'s are taken into account individually in the estimation of e.s.d.'s in distances, angles and torsion angles; correlations between e.s.d.'s in cell parameters are only used when they are defined by crystal symmetry. An approximate (isotropic) treatment of cell e.s.d.'s is used for estimating e.s.d.'s involving l.s. planes.
Refinement. Refinement of *F*^2^ against ALL reflections. The weighted *R*-factor *wR* and goodness of fit *S* are based on *F*^2^, conventional *R*-factors *R* are based on *F*, with *F* set to zero for negative *F*^2^. The threshold expression of *F*^2^ > σ(*F*^2^) is used only for calculating *R*-factors(gt) *etc*. and is not relevant to the choice of reflections for refinement. *R*-factors based on *F*^2^ are statistically about twice as large as those based on *F*, and *R*- factors based on ALL data will be even larger.

### Fractional atomic coordinates and isotropic or equivalent isotropic displacement parameters (Å^2^)

**Table:**

	*x*	*y*	*z*	*U*_iso_*/*U*_eq_	
C2	0.61881 (11)	0.39839 (13)	−0.1020 (3)	0.0241 (5)	
H2	0.5556	0.4068	−0.0918	0.029*	
C4	0.76184 (13)	0.41464 (15)	−0.2179 (4)	0.0308 (5)	
H4	0.8131	0.4368	−0.3039	0.037*	
C3	0.76136 (12)	0.34674 (15)	−0.0291 (4)	0.0303 (4)	
H3	0.8120	0.3132	0.0402	0.036*	
N3	0.54484 (10)	0.08365 (11)	0.8199 (3)	0.0274 (3)	
N2	0.67050 (9)	0.33611 (11)	0.0429 (3)	0.0210 (3)	
N1	0.67229 (10)	0.44554 (12)	−0.2608 (3)	0.0251 (3)	
O1	0.46671 (9)	0.09724 (10)	0.8910 (3)	0.0323 (4)	
O2	0.59895 (10)	0.01721 (12)	0.9069 (3)	0.0406 (4)	
H1	0.6548 (14)	0.4898 (17)	−0.386 (4)	0.030 (5)*	
Cl1	0.88518 (2)	0.09410 (3)	0.84010 (10)	0.02647 (12)	
C8	0.57713 (11)	0.14961 (13)	0.6161 (3)	0.0217 (3)	
C9	0.66889 (12)	0.14545 (14)	0.5571 (4)	0.0303 (4)	
H9	0.7093	0.1022	0.6443	0.036*	
C10	0.69939 (11)	0.20704 (13)	0.3655 (4)	0.0300 (4)	
H10	0.7608	0.2048	0.3216	0.036*	
C5	0.63843 (11)	0.27213 (12)	0.2387 (3)	0.0204 (3)	
C6	0.54623 (11)	0.27679 (13)	0.3038 (4)	0.0264 (4)	
H6	0.5058	0.3212	0.2196	0.032*	
C7	0.51553 (11)	0.21484 (13)	0.4943 (4)	0.0260 (4)	
H7	0.4543	0.2170	0.5397	0.031*	

### Atomic displacement parameters (Å^2^)

**Table:**

	*U*^11^	*U*^22^	*U*^33^	*U*^12^	*U*^13^	*U*^23^
C2	0.0202 (8)	0.0223 (7)	0.0297 (13)	0.0005 (6)	−0.0001 (7)	0.0027 (7)
C4	0.0233 (9)	0.0369 (9)	0.0323 (13)	0.0006 (7)	0.0030 (8)	0.0051 (8)
C3	0.0172 (8)	0.0356 (10)	0.0380 (12)	0.0008 (7)	0.0036 (8)	0.0068 (8)
N3	0.0293 (7)	0.0286 (6)	0.0242 (9)	−0.0071 (5)	−0.0034 (8)	0.0010 (6)
N2	0.0173 (6)	0.0196 (6)	0.0262 (8)	−0.0004 (5)	0.0005 (6)	−0.0007 (6)
N1	0.0247 (7)	0.0232 (6)	0.0274 (8)	−0.0002 (5)	−0.0003 (6)	0.0013 (6)
O1	0.0286 (7)	0.0373 (6)	0.0311 (9)	−0.0060 (5)	0.0047 (6)	0.0014 (6)
O2	0.0347 (7)	0.0438 (8)	0.0432 (10)	−0.0032 (6)	−0.0100 (6)	0.0205 (7)
Cl1	0.01823 (18)	0.02836 (18)	0.0328 (2)	−0.00189 (13)	−0.00026 (19)	0.0061 (2)
C8	0.0231 (8)	0.0202 (7)	0.0219 (9)	−0.0046 (6)	−0.0016 (7)	0.0000 (6)
C9	0.0231 (8)	0.0295 (9)	0.0382 (12)	0.0013 (7)	−0.0040 (8)	0.0089 (8)
C10	0.0161 (7)	0.0326 (8)	0.0415 (12)	0.0018 (6)	−0.0002 (8)	0.0085 (9)
C5	0.0210 (8)	0.0190 (6)	0.0214 (8)	−0.0021 (6)	−0.0001 (6)	−0.0009 (6)
C6	0.0202 (7)	0.0256 (7)	0.0333 (12)	0.0044 (6)	−0.0007 (8)	0.0034 (7)
C7	0.0196 (8)	0.0278 (8)	0.0307 (10)	0.0016 (6)	0.0022 (7)	−0.0005 (7)

### Geometric parameters (Å, º)

**Table:**

C2—N1	1.316 (2)	N1—H1	0.92 (2)
C2—N2	1.343 (2)	C8—C7	1.381 (2)
C2—H2	0.9300	C8—C9	1.381 (2)
C4—C3	1.343 (3)	C9—C10	1.384 (3)
C4—N1	1.382 (2)	C9—H9	0.9300
C4—H4	0.9300	C10—C5	1.388 (2)
C3—N2	1.393 (2)	C10—H10	0.9300
C3—H3	0.9300	C5—C6	1.396 (2)
N3—O1	1.220 (2)	C6—C7	1.382 (3)
N3—O2	1.232 (2)	C6—H6	0.9300
N3—C8	1.474 (2)	C7—H7	0.9300
N2—C5	1.425 (2)		
			
N1—C2—N2	108.78 (15)	C7—C8—C9	122.31 (17)
N1—C2—H2	125.6	C7—C8—N3	119.25 (15)
N2—C2—H2	125.6	C9—C8—N3	118.43 (16)
C3—C4—N1	107.45 (16)	C8—C9—C10	118.58 (16)
C3—C4—H4	126.3	C8—C9—H9	120.7
N1—C4—H4	126.3	C10—C9—H9	120.7
C4—C3—N2	106.90 (16)	C9—C10—C5	120.04 (15)
C4—C3—H3	126.5	C9—C10—H10	120.0
N2—C3—H3	126.5	C5—C10—H10	120.0
O1—N3—O2	124.04 (17)	C10—C5—C6	120.53 (17)
O1—N3—C8	118.59 (14)	C10—C5—N2	119.73 (15)
O2—N3—C8	117.37 (15)	C6—C5—N2	119.73 (15)
C2—N2—C3	107.90 (15)	C7—C6—C5	119.50 (15)
C2—N2—C5	126.14 (14)	C7—C6—H6	120.2
C3—N2—C5	125.95 (15)	C5—C6—H6	120.2
C2—N1—C4	108.96 (16)	C8—C7—C6	119.02 (16)
C2—N1—H1	127.3 (13)	C8—C7—H7	120.5
C4—N1—H1	123.7 (13)	C6—C7—H7	120.5
			
N1—C4—C3—N2	0.1 (2)	C8—C9—C10—C5	0.7 (3)
N1—C2—N2—C3	0.8 (2)	C9—C10—C5—C6	0.4 (3)
N1—C2—N2—C5	179.69 (15)	C9—C10—C5—N2	179.43 (17)
C4—C3—N2—C2	−0.6 (2)	C2—N2—C5—C10	176.61 (17)
C4—C3—N2—C5	−179.45 (15)	C3—N2—C5—C10	−4.7 (3)
N2—C2—N1—C4	−0.7 (2)	C2—N2—C5—C6	−4.4 (3)
C3—C4—N1—C2	0.4 (2)	C3—N2—C5—C6	174.27 (17)
O1—N3—C8—C7	−7.6 (2)	C10—C5—C6—C7	−0.8 (3)
O2—N3—C8—C7	171.90 (17)	N2—C5—C6—C7	−179.81 (15)
O1—N3—C8—C9	171.10 (16)	C9—C8—C7—C6	1.1 (3)
O2—N3—C8—C9	−9.4 (2)	N3—C8—C7—C6	179.81 (16)
C7—C8—C9—C10	−1.5 (3)	C5—C6—C7—C8	0.0 (3)
N3—C8—C9—C10	179.81 (16)		

### Hydrogen-bond geometry (Å, º)

**Table:**

*D*—H···*A*	*D*—H	H···*A*	*D*···*A*	*D*—H···*A*
N1—H1···Cl1^i^	0.92 (2)	2.08 (2)	2.9976 (17)	178 (2)
C9—H9···Cl1	0.93	2.80	3.5898 (19)	144
C2—H2···Cl1^ii^	0.93	2.52	3.4286 (17)	166
C4—H4···O2^i^	0.93	2.29	3.181 (2)	161

Symmetry codes: (i) −*x*+3/2, *y*+1/2, *z*−3/2; (ii) *x*−1/2, −*y*+1/2, *z*−1.
